# How people with dementia and their families decide about moving to a care home and support their needs: development of a decision aid, a qualitative study

**DOI:** 10.1186/s12877-016-0242-1

**Published:** 2016-03-21

**Authors:** Kathryn Lord, Gill Livingston, Sarah Robertson, Claudia Cooper

**Affiliations:** Division of Psychiatry, University College London (UCL), 6th Floor Maple House, London, W1T 7NF UK

**Keywords:** Decision-making, Dementia, Carers, Place of care, Care home

## Abstract

**Background:**

People with dementia and their relatives find decisions about the person with dementia living in a care home difficult.

**Methods:**

We interviewed 20 people with dementia or family carers around the time of this decision in order to design a decision-aid.

**Results:**

Decision-makers balanced the competing priorities of remaining somewhere familiar, family’s wish they remain at home, reduction of risk and effects on carer’s and person with dementia’s physical health. The person with dementia frequently resented their lack of autonomy as decisions about care home moves were made after insight and judgment were impaired. Family consultation usually helped carers but sometimes exacerbated tensions. Direct professional support was appreciated where it was available. There is a need for healthcare professionals to facilitate these conversations around decision-making and to include more than signposting to other organisations.

**Conclusions:**

There is a need for a healthcare professional facilitated decision-aid. This should detail what might change for the person with dementia and their carer, possible resources and alternatives and assist in facilitating discussion with the wider family; further research will develop and test a tool to facilitate decision making about place of care needs.

## Background

People with dementia often need increased care at home or are required to move to live with or nearer relatives or to a 24 h care facility as the illness progresses due to increasing dependency, safety concerns, neuropsychiatric symptoms or family members becoming increasingly frail and unable to care [1]. The decision to move is often difficult and stressful [2, 3], especially as the person with dementia and their family may have never thought that they would live in a care home and there may be disagreement about the need to do so coupled with concerns that the person with dementia may never learn to navigate their new surroundings.

People with dementia may have expressed their wishes about future place of care through advanced statements or family discussions at an earlier time when more able to consider and express their preferences as many people with mild or moderate dementia can participate in decision-making to some extent [4]. In general, when they are well most people do not plan that they or their relatives will move into a care home [3]. Most, who can no longer manage living in their current circumstances, rely on family carers to negotiate choices and make healthcare decisions on their behalf to some extent. A recent systematic review found that family carers find proxy decision-making, especially around place of care, challenging and distressing, especially when decisions are made against the wishes of the care recipient and support from healthcare professionals is lacking [5].

There is limited evidence about how people with dementia and carers make this important and difficult decision and what might reduce their distress. This qualitative study was part of the DECIDE project to create and test a resource to help people with dementia and their family carers make decisions about living arrangements and future place of care. We interviewed both people with dementia and their family carers about how they made these decisions for, to our knowledge, the first time in a published study. We sought to understand the decision-making process, the needs and difficulties they encountered and how these may be overcome.

## Methods

This study was approved by National Research Ethics Service Committee London – Bloomsbury (January 2014; REC Reference: 14/LO/0012).

### Recruitment

Memory clinic clinicians identified and approached potential participants and they were given or sent information sheets. They were approached by the researcher (KL) after a minimum of 24 h and if they were interested they met her, discussed the study and gave written informed consent. If the clinicians or researchers at any point judged any individual lacked capacity as defined and measured by the Mental Capacity Act (2005) [6], they were excluded from participation in the study. Recruitment ceased when it was judged that data saturation was reached; this is when the inclusion of an additional interview did not significantly add to the knowledge that had been gained [7]. Sample size recommendations from the Ottawa guidelines of developing and evaluating patient decision aids were also considered [8].

### Sample

Participants were from memory clinics in inner and outer London and were people with a clinical diagnosis of dementia or their family carers. Eligible carer participants were the current, unpaid, main family or friend carer. In order to encompass a range of views and maximize the validity of our findings we sought to interview a demographically diverse range of people with dementia and carers who were currently in the process of making a decision about, or had recently made the decision about, future place of care or residence. We therefore recruited purposively to include those who lived in inner cities and suburban areas, of either sex, a range of age groups, relationships to the person with dementia, ethnicities and severity of dementia.

We excluded participants who spoke insufficient English to participate in the interview or who did not have the capacity to consent to the interview.

### Interview

All participants were offered the opportunity to be interviewed either alone or, in the case of a person with dementia and carer dyads, to be interviewed together by KL. One of the people with dementia and their family carer were interviewed together. All other interviews were conducted individually and lasted a maximum of one hour.

We conducted semi-structured interviews in participants’ homes, using a topic guide. The topic guide was developed by the authors, based on the findings from the CHOICE study that reported which decisions family carers found difficult to make for people with dementia who did not have the capacity to make their own decisions and facilitators of and barriers to such decisions [3]. As the CHOICE interviews were limited to family members of people with dementia, we then further consulted clinicians from memory clinics (Old Age Psychiatrists and Clinical Psychologists) and dementia researchers to ensure the content of the interviews included any further relevant topics

### Making the decision

We asked people with dementia and carers about current living arrangements; we asked them to discuss they had any concerns about where the person with dementia lives (and prompted them to consider loneliness, distance from family, safety and the care available). We explored any differing views within the family and between the family and the person with dementia. We also asked participants to detail the extent to which the participant had been involved in making decisions, and whether they experienced any difficulties; whether they had felt supported and by whom (with prompts about family and healthcare professionals). We asked whether they had talked to anyone about this decision and if so whether this was helpful.

### Development of a decision aid

We explored with participants what might help make this decision about future place of care and whether there was any information they wish they had had. We then prompted them by showing them a previous information leaflet we had developed based on what had helped family carers called “Deciding about a care home” and asked for any comments and improvements to these in terms of form and content and specifically when and how they would want to use this information.

### Analysis

We digitally audio recorded all interviews and transcribed them verbatim, removing all identifying information from transcripts prior to analysis. We used the qualitative research software Nvivo 9 to code, manage and analyse all data. Two researchers (KL and SR) thematically coded all data independently to ensure reliability, generating a coding frame from initial interviews using a thematic content analytic approach. Disagreements between the researchers were resolved through discussion with each other and a consensus was reached. Participants were offered the opportunity to make any alterations to their own transcripts so that we knew it was a true record of what they intended to say and they were able to elaborate if they wished as a method of quality control and validation. In total only two participants took the opportunity to review their transcripts and made no changes to what they had said.

## Results

### Demographics

We interviewed 20 participants; seven with dementia, four of whom had a family carer also participate and an additional nine other family carers. The socio-demographic details of both carers and people with dementia are detailed in Table 1. The main themes identified are detailed below. One of the participants with dementia had recently moved to a care home due to safety concerns and another had moved out of a care home to their relative’s home in a different part of the country. All of the other five people with dementia interviewed were currently living at home. The nine carers who were interviewed without participation of their relative all had relatives with dementia who were living at home and were considering their options about future place of care.

**Table 1:** Participant characteristics

		Carers (*n* = 13)	People with dementia (*n* = 7)
Sex	Male	4	5
	Female	9	2
Age (years)	Range	32–85	71–87
	Mean	59	79
Relationship to person with dementia	Spouse or Partner	5	-
	Child	7	-
	Niece/Nephew	1	-
Living situation	Alone	2	2
	With partner	10	1
	With other relatives	1	3
	In Care home	-	1
Ethnicity	White British	11	6
	Asian	2	1
Current living environment	Flat/House	13	6
	Residential care home	-	1

#### Who makes the decision?

People with dementia sometimes felt, and resented, that they were not supported to participate in decision-making:

*‘I feel it’s rather humiliating frankly to be treated as incompetent, unable to make my own decisions really’; ‘I feel rather that I’ve sort of been taken over a bit and they do my thinking for me and I don’t really like that very much actually’ [Man with dementia living in a care home; 05]*

There were instances in which people with dementia felt that a change in their living situation was a forced decision over which they had no control or influence. One participant did not think that the person making the decision had the right to do so:

*‘Well I was, quite honestly I was forced into it’; ‘The thing is that I would like to perhaps be given some option’ [referring to son moving him to a care home] [Man with dementia in own home; 04]*

*‘I don’t really feel I’ve given him the rights’ [referring to care home manager getting involved in decision about where to live] [Man with dementia living in a care home; 05]*

In other cases, people with dementia and their carers felt the decision making process had been shared:

*‘Well we all sort of sat around, the three of us [person with dementia and two children] trying to find out where’s the best sort of nursing home’ [Woman with dementia in own home; 08]*

Carers recognized that they were taking over decision making and sometimes found this change of role difficult or overwhelming. They often acknowledged excluding the person with dementia from the process because they lacked insight into the problems necessitating a move:

*‘I’m doing everything what my husband was doing before’ [Wife of person with dementia; 015]*

*‘My whole identity was caring for them’ [Daughter of person with dementia; 06]*

*‘He doesn’t want to go anywhere’ [Wife of person with dementia; 015]*

None of the people with dementia or their family carers described being able to refer to advance statements or written recording of the views of the person with dementia at a time when they did not have such a severe dementia.

#### The wider family

Where carers were making surrogate decisions, such decision-making was often shared with or discussed with the wider family. Moves affected other family members, especially where they were moving from a home shared with their spouse to a care home, or where moves were over longer geographical distances. Sometimes family members were moving to be nearer the person with dementia, other times the person with dementia was moving nearer to their relatives. This was often a difficult decision to make as it ultimately resulted in someone having to leave their home and local area, thus impacting on their social contact outside their immediate family.

*‘The main reason really for wanting to keep her up there [north England] as opposed to bringing her down here [London] to go to a nursing home down here was because of her friends’ [Daughter of person with dementia; 07]*

People with dementia and their families were concerned about isolation of the person with dementia. Sometimes carers were torn between the emotional and practical needs of the person with dementia, their own needs and other family members. One carer excluded the person with dementia to try to protect their feelings:

*‘My father went to pieces when she went into respite [mother with dementia]. This is really important info because that totally changed my view about what could happen to my mother. It made it very clear that if he remained as aware as he is, they couldn’t be separated’ [Daughter of father with dementia; 06]*

*‘The big problem has occurred, as I knew it would, that I don't see anybody’ [Woman with dementia in own home; 08]*

Carers reported consulting family and friends about the decision to move their relative. Some found this helpful whilst others felt it created tension where views differed:

*‘We’re both honest with each other and it’s such a help, I don’t feel I’ve got to hold back or that I’m going to upset her [when talking to another carer of a person with dementia]’ [Wife of person with dementia; 012]*

*‘When I think about it I think there are areas that are still a bit taboo between us, maybe we’re protecting each other’ [Wife of person with dementia; 012]*

#### Familiar environment

All carers expressed a desire to maintain the person with dementia living in their own home and caring for them there where possible as they recognized familiarity of the environment and preservation of daily routine were important:

*‘Well I don’t think either of us will move from here because we’ve been here so long and we like it’ [Husband of person with dementia; 011]*

Proximity to local amenities such as public transport and shops was seen as an important factor in maintaining independence.

The people with dementia valued remaining at home highly, especially as they often lacked insight or did not agree with reasons behind a move:

*‘I have suggested these things to him, that a carer might be satisfactory… well he doesn’t like it… yea because I think it usurped his position’ [Man with dementia in own home; 04]*

Individuals with insight into their dementia often raised the discussion around the need for additional care or moving to a different care setting and expressed feelings of guilt around having the illness.

*‘He feels very guilty anyway… I don’t want him to feel that it’s [the dementia] going to blight my life’. [Wife of person with dementia; 012]*

#### Safety

Safety concerns about falls or unsafe use of gas or electric kitchen devices, coupled with accessibility issues were the most commonly reported problems that triggered the decision making process for both carers and people with dementia:

*‘I think we should be in a flat… having things on one level will help’, ‘I’m keen to be somewhere where we’re not so dependent on a car. So nearer transport, nearer shops’ [Wife of person with dementia; 012]*

*‘I’d like to be sort of more truthful about it you know, not try to kid myself, but there are difficulties. I mean I’d need quite a lot of help I think… the trouble is you see, nobody could be on 24 hours [when discussing carers coming to own home]’ ‘I really think I need to be monitored really’ [Man with dementia in care home; 05]*

In some cases the person with dementia accepted that the carer had concerns and, as they trusted the carer’s view, they also accepted this as a reason to move:

*‘He [the carer] was very much, very keen I should get into somewhere so that I couldn’t fall over’ [Man with dementia in care home; 05]*

#### Physical health

Participants discussed no longer being able to manage at home due to physical health problems. These were unrelated to the dementia but the complexity of problems made solving them difficult:

*‘My brother was taking her [home] and realized there was just no way he could leave her at home, she was just in no state for that [due to pain]’ [Daughter of person with dementia; 07]*

*‘It got very tough because she couldn’t get into the bath due to knee, had to be strip washed’, ‘It’s urgent because she’s falling over’ [Daughter of person with dementia; 06]*

Participants were aware that adjustments to their current environment may enable them to stay at home longer but not all were sure how to make these adjustments and if there were services available to help:

*‘We don’t know who to contact, we are completely lost’ [Wife of person with dementia; 03]*

*‘I still feel that I don’t know all the questions to ask…I mean who gets involved, is it Social Service, who is it? And how does that begin?’ [Wife of person with dementia; 012]*

#### Carer health issues

Several participants, all from the spousal couples interviewed, raised concerns about the carer’s health impacting on their ability to provide care in the future:

*‘I am concerned about [wife’s] health…I do worry that we’re both losing it’ [Man with dementia in own home; 013]*

*‘That is something that worries me, if I get worse, who is going to look after him? That’s my main worry’ [Wife of person with dementia; 015]*

*‘We’ve also got to face the fact that we both might need care’ [Wife of person with dementia; 012]*

#### Uncertainty about the future

A central theme throughout the interviews was the knowledge that dementia progresses but uncertainty about the specific course of the illness:

*‘We’re coping pretty well at the moment but you know in years to come it could, you know it will probably get worse’ [Husband of person with dementia; 011]*

*‘It is one of those things that I should be thinking about more and making more plans about [wife with dementias future living arrangements]’ [Husband of person with dementia; 014]*

*‘I want him to stay here, I want to do as much as I can but I really don’t know what the futures like you know, I have no idea’ [Wife of person with dementia; 015]*

#### Navigating health and social care

Many participants reported being unsure which healthcare professional or agency they should or could talk to, which services they were entitled to or whether services existed:

*‘But we don’t know who to contact, we are completely lost’ [Wife of person with dementia; 03]*

Some felt excluded by inclusion criteria of memory services:

*‘If you are not on medication you are instantaneously discharged from the memory service…. So you are saying the whole service is utterly and totally determined by a pill? And it’s not just that that’s absurd, it’s actually very upsetting, it’s personally, I can’t describe this feeling of exclusion’ [Daughter of person with dementia; 06]*

Lack of support and planning for a crisis from healthcare professionals was highlighted:

*‘One of the problems within the whole decision making process is firstly is it’s very unsupported, but secondly there was no plan b, there was no contingency for a crisis’ [Daughter of person with dementia; 06]*

Nearly all carers and people with dementia expressed concerns regarding how services and care were to be paid for both now and in the future which ultimately impacted on decisions that were made when thinking about place of care:

*‘It was a sense of this can’t carry on and they shouldn’t be bearing the cost of care because they don’t have much savings left and the house is the asset and I don’t know what’s available’ [Daughter of person with dementia; 06]*

*‘Well one thing which I find rather, is that it’s quite expensive, I’m paying £1000 a week’ [for respite accommodation in a care home]… there’s a limit to the amount I could do that’ [Man with dementia living in a care home; 05]*

Previous experiences, both positive and negative, with other family members with dementia who moved to a care home influenced carers and people with dementia interviewed. Media portrayal of care homes also impacted decisions not to use a care home:

*‘What I hear every time on the television, what I read and you know, no, no…we have been married over 50 years and I would hate to put him in a place where he’s not well looked after’ [Wife of person with dementia; 015]*

#### Development of a decision aid

Participants responded positively to the idea of a decision aid focused on future place of care:

*‘It’s probably useful to know that the kind of, what you are thinking yourself is actually the way it is, that’s the way people think, other people, and that’s comforting I think’ [Daughter of person with dementia; 07]*

#### What should be included in a decision aid?

Participants wanted discussions about what services are available at home to be included in a decision aid. In addition, information about changes that may occur for the person with dementia or their carers that may ultimately impact place of care decision-making was sought:

*‘The only thing that I would like to have a bit more of is more information about what help is available at home’ [Wife of person with dementia; 012]*

*‘Probably more of a discussion about the ways in which circumstances could change, how they might change for the carer who’s own health or something may be deteriorating’ [Husband of person with dementia; 014]*

In terms of decision-making about place of care, both people with dementia and carers highlighted the importance that a move to a care home is not the only option available and should not be the sole focus of the information given and sufficient details of alternatives should be provided:

‘*Why is it so much about care homes? Why not have carers living in? … Most people I’ve come across, ok they find it difficult having a carer in their home but it’s still better than being in a care home’ [Daughter of person with dementia; 07]*

Information about how and where to access details about other organisations that may provide support or information about finance or care homes was seen as an important addition to a decision aid. The list of contact details of available resources for carers and people with dementia such as Age UK and the Alzheimer’s Society was well received and provided information participants had not previously had:

*‘Oh how wonderful… Oh yes, excellent, some of these I haven’t heard of!’ [Wife of person with dementia; 012]*

Although these were seen as important, many felt that simply the name and contact information about these organisations was not sufficient and details about what exactly these agencies do would be most welcomed:

*‘It doesn’t tell you what the various organisations, what the resources have to offer… I think you would do better to have a very small number and describe more carefully what they do’ [Husband of person with dementia; 014]*

#### How should a decision aid be delivered?

Participants discussed how the resource could be delivered and the importance of human interaction and support was evident given the complicated nature of this decision-making process:

*‘That moment of being, feeling really supported, that’s why I wonder when you talk about a resource, for me the most important resource are humans… Sitting next to somebody filling in a form together was, I can’t tell you how supportive that was’ [Daughter of a person with dementia; 06]*

The added benefits of the decision aid being delivered by a professional and having the discussion about future place of care can help to clarify views and opinions on the issues that need to be considered in decision-making:

*‘Talking to you has made it clear to me that my responses are very mixed’ [Wife of person with dementia; 012]*

#### When should a decision aid be used?

All participants were very clear that information about future place of care should not be delivered at the point of diagnosis:

*‘I think that will worry people a lot… they will think the worst… let the patient get used to it a little bit, let it sink in a bit you know and see what progress’ [Wife of person with dementia; 015].*

*‘I mean it can’t be right at the beginning, you can’t cope with it’ [Wife of person with dementia; 012]*

Carers felt that waiting until the dementia had progressed and also relying on the knowledge of expertise to raise the issue was important:

*‘Well not on diagnosis, I think you know, maybe after 2 or 3 years’ [Husband of person with dementia; 011]*

*‘That’s probably a judgement of the memory clinic… a major part of the remit of the clinician in the memory clinic you know is to just assess how things are going I think by directly asking the question and also trying to look beyond the answers… people probably are reluctant to say ‘oh it’s all getting a bit much for me’ and perhaps you have to draw that out of them a bit more’ [Husband of person with dementia; 014]*

## Discussion

Our qualitative study was the first to interview people with dementia about their experiences of decision-making around care homes and to interview people at the time of this decision rather than prospectively or retrospectively. The nature of the illness means people with dementia may have had difficulty remembering how decisions were taken; however, where we could, we spoke to carer-care recipient dyads, so we could explore the process from both perspectives. Most of the people with dementia we interviewed did not feel part of decision-making about place of care. Some preferred their family to decide for them but others felt excluded and even humiliated by not being included. Carers often reported that it was not possible to involve the person with dementia in the decision due to their lack of understanding of the issues necessitating a move.

The people with dementia reported a strong desire to continue living in their own homes. Carers recognized and echoed this desire however concerns around the safety and ability of people with dementia were often such that it was not possible. Support both from other family members and healthcare professionals was sought and valued, but consistent with other research reports, many carers found difficulty in negotiating the complicated healthcare system [12]. The deteriorating healthcare status of carers can be a crucial factor in the decision making process.

None of the people interviewed drew on discussions about place of care earlier in the dementing illness or advanced statements when making their decision. Perhaps the dementia was diagnosed too late for the person to be involved in planning care, or perhaps opportunities for discussions that might have eased the difficulty of later decision-making were missed. Carers often find planning difficult, and Advanced Care Plans (ACPs) have not been widely taken up [13]. Decision making for the long term can be avoided due to fear of confrontation with the care recipient and fears of this uncertain future [14]. Sometimes there are too many uncertainties to draw up definitive plans for the future. The unanimity that this planning should not take place near the time of diagnosis certainly reduces the window to make these advance decisions.

We recognize that there may be limitations of the findings due to sample size however in a review of over 500 qualitative research projects sample sizes, the most common were 20 and 30 [15]. We only interviewed people who had capacity, so did not include people with more severe dementia, although we did speak to relatives of people with more severe dementia. We interviewed only those able to speak fluent English so we cannot comment on the needs of those who do not. Ethnicity has been found to affect the decision to look after an individual with dementia at home resulting in presenting later to services, with carers reporting issues with filial piety and obligation (Chang et al., [16, 17]) and that there is often a lack of culturally appropriate facilities (in terms of language and food) [18].

## Conclusions

The decisions about placement were often made at a point in the illness when insight into risks and abilities to stay at home were often lost. Consequently, people with dementia were sometimes unable to contribute fully or at all to the decision, whilst carers felt overwhelmed and distressed. Participants described the decision as a balance between the importance of remaining in a familiar environment and the need to reduce unacceptable levels of risk or accidents at home. The effect on wider family and carers’ health were considered. Support from healthcare professionals was appreciated but sometimes confusing to access or provided inadequate assistance to those making this pivotal decision about place of care for people with dementia. Our findings would support development of an intervention to help people with dementia and their families and carers have facilitated discussions about these issues of future place of care earlier in the illness, when decisions about whether, when and where placement might be needed in future could be shared. Such a resource should be interactive and individual, completed with a healthcare professional so there is opportunity for clarification of thoughts and a written record. It should encourage the use of family and other resources and signpost to these and include what might change for both the person with dementia and their carer and alternatives to care homes. Our findings are not only important for healthcare professionals in terms of how they facilitate conversations with carers but equally important for healthcare commissioners responsible for assigning appropriate amounts of professional resource to provide this level of carer support. Further research should focus on developing and testing a tool for healthcare professionals to facilitate decision-making with carers of people with dementia around future place of care.

## References

[1] Ducharme F, Couture M, Lamontagne J (2012). Decision-making process of family caregivers regarding placement of a cognitively impaired elderly relative. Home Health Care Services Quarter.

[2] Elliott BA, Gessert CE, Peden-McAlpine C (2009). Family decision-making in advanced dementia: narrative and ethics. Scand J Caring Sci.

[3] Livingston G (2010). Making decisions for people with dementia who lack capacity: qualitative study of family carers in UK. BMJ.

[4] Smebye KL, Kirkevold M, Engedal K (2012). How do persons with dementia participate in decision making related to health and daily care? A multi-case study. BMC Health Serv Res.

[5] Lord K, Livingston G, Cooper C (2015). A systematic review of barriers and facilitators to and interventions for proxy decision-making by family carers of people with dementia. Int Psychogeriatr.

[6] Department of Health. The Mental Capacity Act 2005. 2005. http://www.legislation.gov.uk/ukpga/2005/9/contents.

[7] Glaser B, Strauss A (1967). The discovery of grounded theory: Strategies for qualitative research.

[8] O’Connor AM, Jacobsen MJ. *Workbook on Developing and Evaluating Patient Decision Aids Ottawa Hospital Research Institute*. Ottawa: 2003. http://decisionaid.ohri.ca/docs/develop/Develop_DA.pdf

[9] Cooper C, Tandy AR, Balamurali TBS, Livingston G (2010). A systematic review and meta-analysis of ethnic differences in use of dementia treatment, care, and research. Am J Geriatr Psychiatr.

[10] Mahoney R, Regan C, Katona C, Livingston G (2005). Anxiety and depression in family caregivers of people with Alzheimer disease: the LASER-AD study. Am J Geriatr Psychiatry.

[11] Miles M, Huberman A (1994). Qualitative data analysis: an expanded sourcebook.

[12] Graneheim UH, Johansson A, Lindgren BM (2014). Family caregivers’ experiences of relinquishing the care of a person with dementia to a nursing home: insights from a meta-ethnographic study. Scand J Caring Sci.

[13] Dening KH, Jones L, Sampson EL (2012). Preferences for end-of-life care: A nominal group study of people with dementia and their family carers. Palliat Med.

[14] Sampson MS, Clark A (2015). Deferred or chickened out?’ Decision making among male carers of people with dementia. Dementia.

[15] Mason M (2010). Sample size and saturation in PhD studies using qualitative interviews. Forum Qual Sozialforschung / Forum Qual Soc Res.

[16] Chang YP, Schneider JK (2010). Decision-making process of nursing home placement among Chinese family caregivers. Perspect Psychiatric Care.

[17] Chang YP, Schneider JK, Sessanna L (2011). Decisional conflict among Chinese family caregivers regarding nursing home placement of older adults with dementia. J Aging Stud.

[18] Caldwell L, Low LF, Brodaty B (2014). Caregivers’ experience of the decision-making process for placing a person with dementia into a nursing home: comparing caregivers from Chinese ethnic minority with those from English-speaking backgrounds. Int Psychogeriatr.

